# Individualized surgical treatment of giant tuberculum sellae meningioma: Unilateral subfrontal approach vs. endoscopic transsphenoidal approach

**DOI:** 10.3389/fsurg.2022.990646

**Published:** 2022-09-05

**Authors:** Yang Li, Chao Zhang, Jun Su, Chaoying Qin, Xiangyu Wang, Yue Li, Qing Liu

**Affiliations:** ^1^ Department of Neurosurgery in Xiangya Hospital, Central South University, Changsha, China; ^2^ Department of Neurosurgery, The First Affiliated Hospital of Zhejiang University, Hangzhou, China; ^3^ Department of Neurosurgery, Hunan Children’s Hospital, Changsha, China

**Keywords:** tuberculum sellae meningioma, endoscopic transsphenoidal approach, unilateral subfrontal approach, skull base surgery, visual function

## Abstract

**Objective:**

Giant tuberculum sellae meningiomas (TSMs) are deeply located in the suprasellar region and extensively compressed or encased in the surrounding neurovascular structures, making gross total resection (GTR) without postoperative visual impairment challenging. The authors presented individualized unilateral subfrontal approach and endoscopic transsphenoidal approach (ETSA) in a series of patients and elaborated on their advantages and indications in resecting giant TSMs.

**Methods:**

A total of 38 patients with giant TSMs operated by a single surgeon between March 2012 and November 2021 were retrospectively reviewed. Patients underwent unilateral subfrontal approach and ETSA according to preoperative imaging characteristics. Tumor characteristics, surgical details, preoperative symptoms, and neurological outcomes of TSMs patients were collected and analyzed.

**Results:**

In 31 patients operated with the unilateral subfrontal approach, total resection (Simpson grade I or II) was achieved in 27 patients (87.0%), while 6 patients (85.7%) achieved GTR in 7 patients using ETSA. The postoperative visual improvement was maintained in 22 (81.5%) and 5 patients (83.3%). Recurrence or progression was only observed in 2 (7.4%) patients operated with the unilateral subfrontal approach. There was no mortality in our series.

**Conclusions:**

Preoperative imaging and visual function are important for surgical approach selection. Maximum tumor resection and optic nerve protection can be achieved concurrently by taking advantage of these surgical approaches. The cerebral artery protection strategies and individualized surgical techniques provide great utility in improving a patient's quality of life.

## Introduction

As one of the most common meningioma in the skull base, tuberculum sellae meningiomas (TSMs) account for 5%–10% of intracranial meningiomas (1). TSMs originate from the dura of the tuberculum sellae, chiasmatic sulcus, and limbus sphenoidale (2). These tumors were located in the suprasellar space and tend to compress the optic chiasm and optic nerves. Most of the patients were presented with progressive vision loss and visual field defects (3, 4). Except for nerves/chiasm, giant TSMs (maximum diameters ≥3 cm) usually encase vital neurovascular structures, including the carotid artery, forebrain arteries, and pituitary gland–making gross total resection (GTR) of the tumor while preserving neurological functions challenging (5, 6). The postoperative visual deterioration rates from 2.1% to 44%, and GTR rates range from 65% to 90% (7–10). However, due to the limitation of stereotactic radiotherapy, three-dimensional conformal radiotherapy, and modulated radiation therapy (10, 11), microsurgical resection is still the primary treatment for TSMs.

The primary goal of the TSMs surgery was achieving gross total resection while preserving optic nerve functions during the past few decades. Multiple transcranial microsurgical approaches have been developed to achieve this goal, including the frontolateral approach, pterional approach, lateral supraorbital approach, and unilateral approach (9, 12–15). However, with advances in endoscopy and the development of innovative strategies in the expanded endonasal approach (16, 17), the endoscopic transsphenoidal approach (ETSA) seems to have gained more acceptance in the surgical treatment of TSMs (18–20). ETSA offered a better visualization, minimized the additional surgical damage, and avoided the contraction of the optic nerve during TSM surgery (21, 22). However, there have been many controversies over indications for ETSA, especially in the surgical resection of giant TSMs.

According to recent reports and our experiences, giant TSMs were defined as meningiomas larger than 3 cm in at least one of the three-dimensional planes and extended laterally on either side of the internal carotid arteries or optic nerves (7). Giant TSMs tend to invade the adventitia of the anterior cerebral artery (ACA), extended laterally to the internal carotid artery (ICA), eroded the bone of the skull base, and calcified central tumor zone. These characteristics make gross total resection of TSMs *via* ETSA challenging and entail greater risk of artery and nerve injury. Thus, transcranial approaches became the preferred method for surgical treatment giant TSMs.

In this research, we reviewed the characteristics and outcomes of 38 consecutive patients with giant TSMs operated by the senior author (Qing Liu). We described our experience in selecting unilateral subfrontal approach or ETSA for the surgical treatment of giant TSMs. We specifically evaluated the advantages of the two approaches and proved that individualized surgical strategies provide a better prognosis for patients.

## Materials and methods

### Study design

Between March 2012 to November 2021, 38 consecutive patients diagnosed with TSMs were retrospectively analyzed. The patients who participated in this study were operated on by the senior author (Qing Liu) using the unilateral subfrontal approach and ETSA at the Department of Neurosurgery, Xiangya Hospital, Central South University. Only meningiomas not less than 3 cm in at least one of the three-dimensional planes and originated from the tuberculum sellae were included in this study. Neuroimaging, intraoperative video, and neurofunctions were recorded and analyzed. Meningiomas originating from the diaphragm sellae, anterior clinoid, cavernous sinus, and anterior skull base with secondary involvement of the parasellar region were excluded.

### Evaluation of tumor characteristics

Tumor characteristics, including tumor dimension, depth of the sella turcica (ST), the angle between the planum sphenoidale (PS) and ST, and tumor extension were evaluated by preoperative contrast-enhanced magnetic resonance imaging (MRI) and computed tomography angiography (CTA), which were confirmed by intraoperative observation. To evaluate the relationship between tumor and surrounding vascular structures, patients underwent contrast-enhanced MRI and CTA before surgery. Visual functions were evaluated at the ophthalmology department of our institute.

### Unilateral subfrontal approach

**Figure 1:**
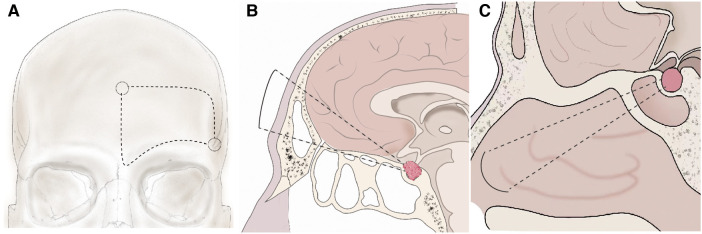
Schema of the unilateral subfrontal approach and ETSA. (**A**) The bone flap using in the unilateral subfrontal approach. One key burr hole (arrow) was placed behind the frontozygomatic process and below the superior temporal line. The other was placed in the midline above the nasion. (**B**) After unilateral subfrontal craniotomy, extradural dissection was performed in the central portion of the anterior cranial fossa around the cribriform plate. Elevation of the subfrontal dura from the planum sphenoidale can access tumor extension superior to the suprasellar region. (**C**) After drilling the bone of the sella, planum sphenoidale, and the tuberculum sellae, the dura was exposed and coagulated. The tumor in the sellar and suprasellar can be removed under endoscopic vision.

The unilateral subfrontal approach provides a larger field of vision to resect large TSMs than ETSA and the indications for the approach include: (1) the preoperative images indicated that the tumor extended to the optic canal; (2) the tumor was calcified and resulted in stenosis of cranial arteries; (3) tumor extended laterally to the ICA; (4) the ICA, ACA and their perforating branches were encased extensively by the tumor; and (5) the extensive calcification of the skull base (tuberculum sella, anterior clinoid, and anterior skull base), which need to drill the involved bone and skull base reconstruction. Patients were positioned supine, and their heads were rotated 20°–30° to the contralateral side and retroflexed 10°–20° to reduce frontal lobe retraction. The incision of the unilateral subfrontal approach was initiated above the palpated zygoma and continued superiorly behind the hairline toward the limit of the contralateral hairline. To avoid injury to the frontal branch of the facial nerve, the subgaleal scalp along with the temporal muscle is split and retracted toward the zygomatic arch. The bone flap was made by using the craniotome, and the superciliary arch can be identified and act as the baseline of the bone flap (Figure 1A). A temporal craniotomy was added when the tumor extended laterally. Under microscopic view, the dura mater was cut, followed by elevating the frontal lobe using a self-retaining brain retractor (Figure 1B). Tumor debulking was performed after identification of ICA, an optic nerve encased by the tumor. Dissection proceeded from the ICA to the ACA and anterior communicating artery (AComA). The arachnoid plane between the tumor and the optic nerve or artery should be maintained carefully because it can act as a barrier to protect the perforating branches and blood supply of the optic nerve. The residual portion of the tumor was removed in a piecemeal fashion using a microscissor and bipolar forceps. Only when the tumor was too hard for resection using an ultrasonic surgical aspirator or invade the adventitia of the ICA, we must leave the residual tumor in this area and then perform postoperative stereotactic radiotherapy. All hyperostotic bone was removed with a high-speed drill. The dural attachment of the tumor was resected and coagulated. The tuberculum sellae, planum sphenoidale, and frontal sinus were then tamponaded with bone wax and covered with part of the subgaleal scalp and the temporal muscle in case of cerebrospinal fluid leakage. The extent of tumor resection was evaluated according to the Simpson grading scale (23).

### Endoscopic transsphenoidal approach

ETSA was also applied to the patients with giant TMSs, significantly when the tumor extended superiorly to the anterior skull base or inferiorly to the sellar region but was limited to the medial of the optic nerve. The indications for ETSA were as follows: (1) extension of the tumor limited in the sella or suprasellar region; (2) tumor growth in the medial and inferior sides of the optic nerve; (3) tumor was soft and loosely adherent to the arteries or optic nerve; (4) tumor base centered around tuberculum sella without extensive extension; (5) The angle between PS and the ST was smaller than 90° (Supplementary Figure S1); and (6) The depth of the ST was larger than 1 cm (Supplementary Figure S1). The patients were positioned supine and their heads were fixed by a Mayfield headrest. Under the endoscopic vision, the sphenoid sinus and the sphenoid ostia were identified (Figure 1C). The nasoseptal flap should be dissected and preserved carefully. Then, the middle and superior turbinate were exposed and resected. Drilling of the bone started from the sella, followed by the planum sphenoidale, and finally the tuberculum sellae. Before opening the dura, the position of the ICA must be confirmed again by endoscopic visualization. The dura below and above the diaphragma sellae was cut and coagulated. Extensive coagulation was performed to reduce the extradural blood supply of the tumor. To reduce tumor volume, the tumor base was debulked and removed in small pieces by dissection. The arachnoidal dissection plane between the tumor and the optic nerve or ACA should be established and maintained, as the plane was necessary for efficient total resection. In particular, the pituitary gland and stalk which can be found at the posterior margin of the tumor should be preserved and dissected carefully. A 30° view angle endoscope was used to find and remove residual tumors extended beyond the surgical field. After the tumor was removed completely, the skull base defect was firstly repaired with an artificial dura mater, then covered by a vascularized nasoseptal flap, and the nasal cavity was packed with Vaseline gauze finally.

### Vascular protection strategies

Preoperative CTA indicated that giant TSMs tend to compress, dislocate, or even encase multiple intracranial arteries, including ICA, ACA, AcomA, middle cerebral artery (MCA), and basilar artery (BA). In most cases, the ICA and MCA were dislocated posterolaterally, and the AcomA and BA were dislocated posteriorly. With the help of preoperative imaging, the localization of the dislocated or encased arteries can be identified easier. Furthermore, cranial artery stenosis proved by preoperative CTA usually indicated that the adventitia was invaded by the tumor, making gross total tumor resection entail a huge risk of artery injury.

After opening the dura mater, the ipsilateral ICA should be identified first. Then, the arachnoidal dissection plane between ICA and the tumor should be followed and maintained. The plane can act as a physical barrier to protect ICA against operative injury. The arterial supply of the chiasm and optic nerve mainly arises from the superior hypophyseal arteries, which course through the inferior surface of the optic nerves. Thus, dissection of the tumor beneath the optic nerve entails a greater risk of superior hypophyseal artery injury. Superior hypophysial artery (SHA) arising from the ICA trunk and pushed backward by the tumor. Efforts must be made to dissect the artery from the tumor to prevent pituitary disorder. When the tumor invaded the adventitia of intracranial arteries, the arachnoidal plane between the tumor and artery was interrupted. Under this circumstance, aggressive tumor resection might injure the artery. The residual tumor was left in this area and treated with postoperative stereotactic radiotherapy.

### Management of the optic nerve

TSMs impair vision acuity or visual field by directly compressing optic nerves and optic chiasm or decreasing the vascular supply of the optic nerves and optic chiasm. Thus, vulnerable optic nerves and optic chiasm entail a higher risk of intraoperative injury. Preoperative MRI images were used to estimate the relationship between tumor and optic nerve. Through the images, we can also find TSMs usually located in the suprasellar region with extending to the ipsilateral optic canal, dislocating the optic chiasm posteriorly and the optic nerves laterally. Thus, we can easily confirm the localization of neurovascular structures, such as optic nerves and chiasm. When tumors invade into the optic canal extensively, we sectioned the falciform ligament and drilled the roof of the optic canal at the very beginning of the surgery. Because the sharp margin of the falciform ligament may result in severer intraoperative injury of the optic nerve. Furthermore, opening the optic canal enlarged the space for resection of the tumor invading into the optic canal.

To improve the visual acuity of TSMs patients, the blood supply of the optic nerve and optic chiasm should be protected carefully. However, giant TSMs commonly extend inferomedially to the optic nerve or even encase it. The arterial supply of the chiasm and optic nerve mainly distally along the inferior surface of the optic nerves, dissection of the tumor must remain in the arachnoid plane intact and proceed on the superior surface of the optic nerve. Ophthalmic artery arising from the ICA trunk and coursing on the superior surface of the canalicular segment of the optic nerve. To avoid injury, all perforating arteries in the optic canal and orbit must be preserved. In conclusion, optic canal unroofing, preservation of the blood supply, and dissection along the arachnoid plane increase the rate of improved postoperative optic nerve function.

## Result

### Patient population

A total of 38 consecutive patients (15 males and 23 females) diagnosed with giant TSMs (maximal diameter ≥3 cm) were retrospectively analyzed. The median age was 47.7 years for all patients. The unilateral subfrontal approach was used in 31 patients, while the ETSA was applied in seven patients. The average length of surgery was longer for ETSA (6.3 h) compared with the unilateral subfrontal approach (4.2 h). However, the length of hospital stays in patients performed with the ETSA (4.7 days) was shorter than the unilateral subfrontal approach (7.3 days). The most common presenting symptoms were visual impairment (89.2%), visual field defect (65.8%), and headache (52.6%). The Karnofsky Performance Scale (KPS) was used to assess the life quality of the patients, and the mean KPS was 71.4 ± 7.4 preoperatively. All the removed meningiomas were confirmed by pathological examination.

### Tumor characteristics

TSMs originate from the dura of the tuberculum sellae, chiasmatic sulcus, limbus sphenoidale, and growing upward in the suprasellar region were included. Through preoperative imaging and intraoperative observation, we found that giant TSMs often compress or encase the neurovascular structures of the sellar, suprasellar, and parasellar region extensively, including the optic nerve, optic canal, pituitary gland, anterior clinoid, cavernous sinus and so on (Table 1). Optic nerve and optic chiasm compression were exhibited in almost all patients (31 and 29 patients) with giant TSMs. By contrast, the number of tumors that extend laterally to the sphenoid ridge or cavernous sinus lateral wall was only observed in nine patients. Similarly, tumor calcification and edema were only found in 12 and 6 patients (Table 1). We can conclude that in patients operated with the unilateral subfrontal approach, TSMs usually invaded extensively the planum sphenoidal, suprasellar and parasellar region, and optic canal, and the angle between PS and ST was larger than 90°. However, in patients operated with ETSA, the tumor growth was limited in the suprasellar and sellar region and the consistency was softer, but the depth of the ST was larger. In conclusion, we were able to choose which surgical approaches could be performed by evaluating the above characteristics.

**Table 1:** Clinical characteristics of patients with giant TSMs.

Characteristics	No. of patients	
	Subfrontal approach	ETSA
Tumor dimension, median (cm)	3.7	3.1
Depth of the sella turcica (cm)	1.2	1.9
The angle between PS and ST (°)	101.1	88.5
Planum sphenoidal extension	29	3
Tuberculum sellae hyperostosis	10	3
Sellar region extension	25	7
Pituitary gland involvement	30	6
Optic nerve compression	31	7
Optic chiasm compression	29	6
Optic canal involvement	9	0
Intraorbital extension	6	0
Cavernous sinus involvement	9	0
Sphenoid ridge extension	9	0
Tumor calcification	12	0
Tumor Consistency (hard)^a^	13	1
Brain edema	6	0

ETSA, endoscopic transsphenoidal approach; ST, sella turcica.

^a^ Tumor consistency was defined as hard when the tumor could not be aspirated even with an ultrasonic surgical aspirator.

### Cranial artery involvement

The preoperative imaging and intraoperative observation indicated the involvement of the tumor with the ICA, ACA, AcomA, and MCA. According to Romani's classification (15), we classified the relationship between tumor and arteries into attachment, dislocation, and encasement based on preoperative imaging and operative observation. Giant TSMs mainly involve ICA, ACA, AcomA, and its perforating branches (SHA), whereas MCA was only involved in a few large TSMs which extend laterally (Table 2). We can also conclude that when the tumor was extended laterally and involved in the MCA, only the unilateral subfrontal approach can be applied to achieve total resection.

**Table 2:** The relationship between the giant TSMs and cerebral arteries.

Artery	Classification	Subfrontal approach		ETSA		Total No. (%)
		Unilateral	Bilateral	Unilateral	Bilateral	
ICA	Attachment	5	10	1	3	19 (50)
	Dislocation	13	5	3	1	22 (57.9)
	Encasement	8	2	0	0	10 (26.3)
ACA	Attachment	7	3	1	1	12 (31.6)
	Dislocation	5	12	2	3	22 (57.9)
	Encasement	2	9	1	1	13 (34.2)
AcomA	Attachment	5		2		7 (18.4)
	Dislocation	17		3		20 (52.6)
	Encasement	9		2		11 (28.9)
MCA	Attachment	2	3	0	0	5 (13.2)
	Dislocation	5	7	0	0	12 (31.6)
	Encasement	3	2	0	0	5 (13.2)
SHA	Attachment	4	2	1	1	8 (21.1)
	Dislocation	8	13	1	3	25 (65.8)
	Encasement	4	8	0	2	14 (36.8)

ICA, internal carotid artery; ACA, anterior cerebral artery; AcomA, anterior communicating artery; MCA, middle cerebral artery; SHA, superior hypophysial artery.

### Surgical results

The extent of tumor resection was evaluated by the Simpson grading scale. Total resection (Simpson grades I and II) was achieved in 33 patients. Subtotal resection (Simpson grade III) and partial resection (Simpson grade IV) were achieved in 3 and 2 patients, respectively (Table 3). We found there was no significant difference in total removal of the tumor when compared with the unilateral subfrontal approach and the ETSA (*p* > 0.99), but this result still needed to be proven in a multicenter study. In patients performed with the unilateral subfrontal approach, two patients achieved Simpson III grade resection because of adventitia invasion, and two achieved Simpson IV owing to the tumor calcification and densely adherent to the ICA and ACA. In patients performed with ETSA, Simpson III grade resection was achieved in 1 patient because the tumor grew laterally over the optic nerve, but the preoperative MRI missed it.

**Table 3:** The extent of resection in patients using different surgical approaches.

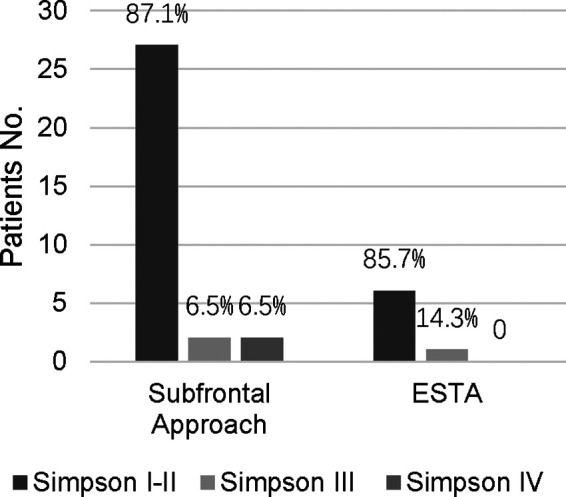

### Neuroophthalmological outcome

Visual deficit was present in 32 patients preoperatively (Table 4). Among all 38 patients, 31 patients were treated with the unilateral subfrontal approach, the rest 7 patients were treated by ETSA. The visual function of most patients (25 patients) improved or remained stable after the surgery. Only a few patients (6 patients) suffered from postoperative visual deterioration. There was no difference in visual improvement between the subfrontal approach and the ETSA in our series (*p *= 0.984), which was different from other studies that have shown more visual improvement with the ETSA.

**Table 4:** Results of visual acuity in relation to tumor size.

Visual symptoms	No. of patients		*p*-value
	Subfrontal approach	ETSA	
Preop			
Yes	27	5	0.302
No	4	2	
Postop			
Improved	15	4	0.984
Worsened	6	0	
Stable	10	3	

ETSA, endoscopic transsphenoidal approach.

### Tumor recurrence and follow up

All 38 patients were followed up regularly by the first author at 6-month intervals for the first year, and annually thereafter. Follow-up was performed with contrast-enhanced MRI and clinical status. The actual follow-up time ranges from 6 to 120 months (mean, 66.1 months). The mean KPS evaluated in patients at follow-up was 86± 7.7 months. Five patients with residual tumors received stereotactic radiotherapy 3 months after the surgery. There was no recurrence at the follow-up.

## Discussion

In this study, we have elaborated the surgical techniques of the unilateral subfrontal approach and ETSA, which can be applied to the individualized surgical treatment of giant TSMs. Preoperative imaging was performed routinely to guide individualized surgical strategy making. Instead of being limited to one single surgical approach, we applied the unilateral subfrontal approach and the ETSA to 38 patients with giant TSMs according to their tumor characteristics. Owing to making full use of the advantages of the subfrontal approach and the ETSA, the GTR of giant TSMs was up to 86.8%, while the postoperative optic nerve dysfunction rate was only 15.8%.

**Figure 2:**
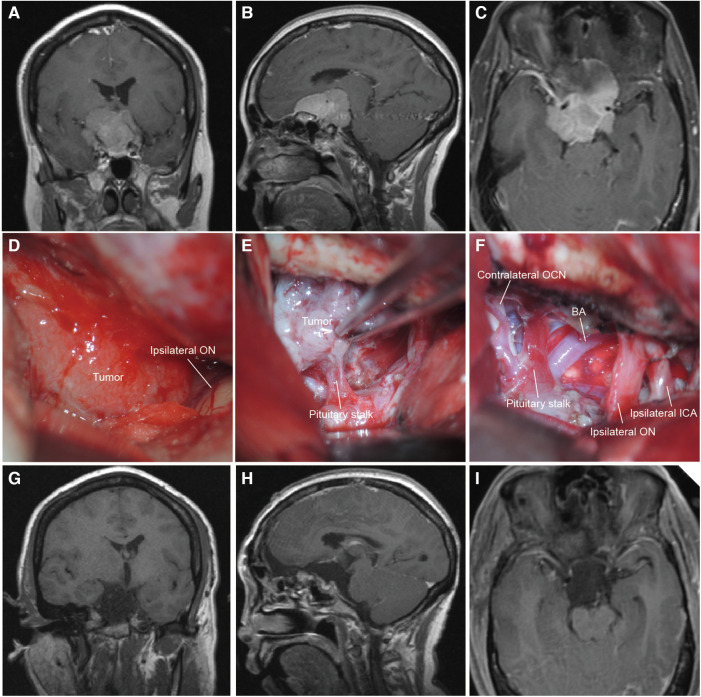
The MRI and intraoperative view of a patient operated with the unilateral subfrontal approach. (**A–C**) Preoperative T1-weighted MRI scan showing the giant TSM (maximal diameter = 4.4 cm) extended lateral to the ICA and encased neurovascular structures of the suprasellar region. **(D,E**) Intraoperative microscopic view showing the tumor through the right bilateral subfrontal approach. The ipsilateral optic nerve was displaced laterally and the pituitary was encased by the tumor. (**F**) Intraoperative microscopic view after total tumor resection. The ipsilateral ICA and optic nerve were preserved. (**G–I**) Postoperative T1-weighted MRI scan confirming total tumor removal. BA, basilar artery; OCN, oculomotor nerve; ICA, internal carotid artery.

In 1916, Cushing performed the first total surgical resection of TSM (1) and operated on 24 cases of tuberculum sellae meningiomas within 20 years. Since then, the transcranial approach has been the primary treatment for TSM. Nowadays, multiple transcranial approaches have been developed and applied to the surgery of TSM, such as the pterional approach (1, 14, 24), lateral supraorbital approach (15), subfrontal approach (6, 25), and interhemispheric approach (26). However, there were still many controversies about which approaches can be applied to achieve GTR better while improving the optic nerve function. For example, the pterional approach can visualize the ICA and minimize the retraction of the frontal lobe easily, but with narrow space and angle and a higher risk of profuse bleeding (14). In most of our surgical series, the unilateral subfrontal approach was the primary surgical approach applied to resect giant TSMs (Figure 2). Compared with other traditional surgical approaches, the unilateral subfrontal approach provides a better medial view of the suprasellar region and is more flexible to achieve GTR when the tumor extends laterally or upward. For instance, when the tumor is extended laterally, the unilateral subfrontal approach combined with the pterional approach allowed us access to the sellar, suprasellar, and parasellar region to achieve GTR easily.

With the advancement of endoscopic technology, which provides better visualization and less invasion during operation, it is now possible to safely resect TSMs by ETSA. Hae-Dong Jho performed the first total surgical resection of TSM by a pure endoscopic approach in 2001 (27). Since then, the ETSA has been developed and widely applied to the surgical treatment of TSMs (28–30). In theory, ETSA avoids some surgical complications by approaching the TSM through the dura base, thereby minimizing the contraction of the frontal lobe, olfactory nerve, and optic nerve. Therefore, the postoperative visual loss rate in patients with ETSA was only 1.3%, compared with 9.2% in patients operated transcranially (31). For patients, the ETSA was minimally invasive, more comfortable, and hospital stay were often shorter (32, 33). The ETSA also provided a clear visualization of perforators of the optic nerve and pituitary stalk, making preservation of the blood supply of the optic nerve and pituitary gland possible (34). In conclusion, ETSA has multiple advantages in the surgical treatment of TSMs (maximal diameters <3 cm). However, multiple problems still need to be solved when applying the ETSA to the treatment of giant TSMs (maximal diameters ≥3 cm). For example (1) giant TSMs extend over the optic nerve to the optic canal or above and lateral to the anterior clinoid process cannot be resected; (2) the incidence of cerebrospinal fluid leakage was high as 20%–30% (28); (3) tumor grow laterally and densely adherent or invade the cavernous; (4) patients have critical basic diseases and cannot bear long time operation.

**Figure 3:**
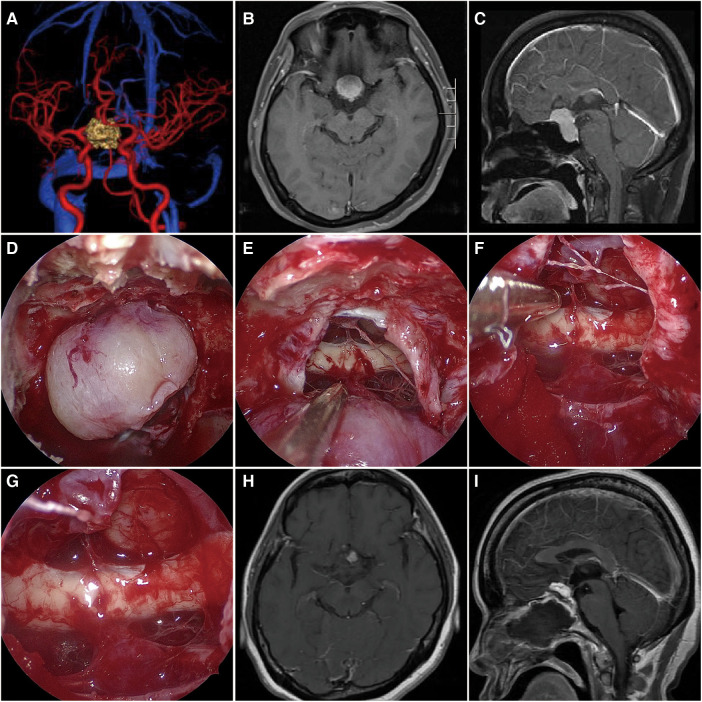
The MRI and intraoperative view of a patient operated with the ETSA. (**A**) Preoperative CTA has shown the bilateral ICA and ACA were compressed by the tumor. (**B,C**) Preoperative T1-weighted MRI scan showing the giant TSM (maximal diameter = 3.1 cm) growth limited in the bilateral ICA. (**D,E**) Intraoperative microscopic view showing the tumor and the optic chiasm after opening the dura. (**F,G**) The tumor was totally removed, while the bilateral optic nerve and pituitary stalk were preserved. **(H,I)** Postoperative T1-weighted MRI scan confirming tumor removal.

To achieve maximal tumor resection and preservation of visual function Indications, we applied individualized surgical approach for patients with TSM based on preoperative imaging characteristics. For example, when the angle between the PS and ST was less than 90° or the depth of the sella turcica larger than 1 cm, the unilateral subfrontal approach was difficult to remove the tumor invaded into the sellar region and making total resection impossible, we would choose the ETSA for tumor resection. However, when we found the tumor extended extensively and invaded into the arteries, the unilateral subfrontal approach was the primary approach for tumor removal. In 31 giant TSMs patients operated through the subfrontal approach, total tumor removal (Simpson grades I and II) was achieved in 27 patients. Only five patients suffered from postoperative brain edema, and six from visual acuity deterioration. In our series, ETSA only applied to seven giant TSMs (Figure 3). Among seven patients who underwent ETSA, total tumor resection (Simpson grades I and II) was achieved in six patients. The postoperative visual improvement was observed in four cases. The mean in-hospital day was only 4.7 days, compared with 7.3 days for patients who underwent the subfrontal approach. However, the risk of pituitary dysfunction and cerebrospinal fluid leakage may be higher in patients undergoing ETSA. These complications may result from excessive manipulation of the pituitary gland during sphenoidal bone removal and injury of the blood supply of the pituitary gland.

Postoperative visual deficits were both found in patients who underwent the subfrontal approach and the ETSA, but the visual deficits of the two approaches were not statistically significant. According to large-scale reports, the ETAS seems to be the primary approach to achieving better visual outcomes in the surgical treatment of TSMs (27–29). The vision improvement rate ranged from 59% to 87% in patients who underwent the ETSA, but the rate was only 25%–61% in patients who underwent the transcranial approach (27–29). However, the vision improvement rate was not statistically different in our series. This difference may be attributed to tumor size, as we only included the giant TSMs (maximum diameters ≥3 cm) in our research. In our experience, giant TSMs tend to densely compress the optic nerve and interrupt the blood supply of the optic nerve, making the deterioration of visual function irreversible. Similarly, Rosenstein and Symon proved that tumors smaller than 3 cm had a better visual outcome compared with those larger than 3 cm (30). The same results were found in other series with tumors larger than 3 or 4 cm in diameter (15, 31). In general, only six patients (15.8%) suffered from postoperative deterioration, while the visual functions were improved or stable in most of the patients (84.2%). We attribute these to vascular protection strategies. In conclusion, maximal tumor GTR and visual function improvement can be achieved by taking full advantage of the benefits of the unilateral subfrontal approach and the ETAS.

## Conclusion

There was no significant difference in GTR rate and vision outcome between the unilateral subfrontal approach and the ETAS for resection of giant TSMs. By making individualized surgical approaches, surgical complications and hospital stays of patients were reduced. Furthermore, maximal resection and preservation of visual function can be achieved by performing individualized surgical strategies.

### Limitation of the study

The patients involved in this study were operated on by a single surgeon and institution. Prospective studies and multi-organizational research with larger sample sizes are still needed to demonstrate the feasibility of the surgical techniques mentioned in this study.

## Data Availability

The original contributions presented in the study are included in the article/Supplementary Material, further inquiries can be directed to the corresponding author/s.

## Supplementary material

Supplementary Figure 1 Evaluation the angle between the panum sphenoidal and the depth of the sella turcica in a TSM patient. Red line: angle between the panum sphenoidal and the sella turcica; blue dotted line: the line between the planum sphenoidal clivus; blue line: the depth of the sella turcica.
