# Supplementary figures and images for: Individualized surgical treatment of giant tuberculum sellae meningioma: Unilateral subfrontal approach vs. endoscopic transsphenoidal approach
Source: Front Surg. 2022 Sep 5;9:990646. doi: 10.3389/fsurg.2022.990646 (PMC9890549; doi:10.3389/fsurg.2022.990646)

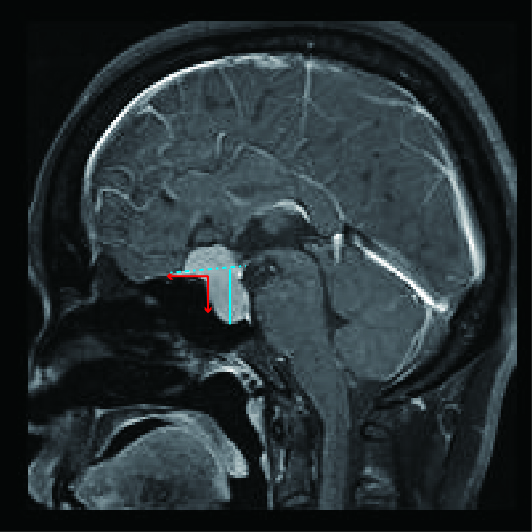

Supplement: Supplementary Figure 1 — Evaluation the angle between the panum sphenoidal and the depth of the sella turcica in a TSM patient. Red line: angle between the panum sphenoidal and the sella turcica; blue dotted line: the line between the planum sphenoidal clivus; blue line: the depth of the sella turcica. [file Image1.jpeg]
